# Neonatology: Topics on Puppies and Kittens Neonatal Management to Improve Neonatal Outcome

**DOI:** 10.3390/ani12233426

**Published:** 2022-12-05

**Authors:** Keylla Helena Nobre Pacífico Pereira, Kárita da Mata Fuchs, Jaqueline Valença Corrêa, Simone Biagio Chiacchio, Maria Lucia Gomes Lourenço

**Affiliations:** Veterinary Neonatology Research Group, Department of Veterinary Clinic, School of Veterinary Medicine and Animal Science, São Paulo State University (UNESP), Botucatu 18618-970, SP, Brazil

**Keywords:** neonatal health, kennel, cattery, management, mortality

## Abstract

**Simple Summary:**

Breeders still see high rates of neonatal mortality in their kennels and catteries globally, which makes the perinatal period a challenge for veterinarians and breeders. This review describes a wide variety of factors that can potentially affect the health of a litter during pregnancy, birth, and the first few weeks of life, which requires both the veterinarian and the breeder to have knowledge of maternal and neonatal care. Knowledge in neonatology is essential for correct management and preventive measures in breeding, impacting well-being and greater neonatal survival.

**Abstract:**

The productivity of kennels and catteries is directly linked to maternal prolificacy and neonatal survival. During the neonatal period, mortality is high, not only due to environmental factors after birth but also with regard to gestational fetal life, complications during delivery, and management errors. Neonatology is an area of veterinary medicine where having a strong knowledge base of applied physiology and common clinical presentations for newborns can often favor better outcomes and greater neonatal survival. The teaching of animal theriogenology topics, particularly neonatal medicine, in the veterinary curriculum has evolved significantly in recent years. It is essential that the veterinarian understands the maternal and neonatal particularities and the main aspects involved in the handling of puppies and kittens for the instruction of the correct handling to the breeders and better medical conduct. The breeder of dogs and cats, experienced or not, must count on the help of a veterinarian in their breeding. Proper management, constant monitoring of the litter, and prompt assistance are the keys to successful breeding.

## 1. Introduction

The neonatal period in dogs and cats is still a major challenge for veterinarians and breeders, as it is associated with high rates of morbidity and mortality globally, ranging from 5.7 to 35% in dogs [1,2,3,4,5,6,7] and from 14 to 16% in cats [8,9,10].

There are a wide variety of factors that can potentially affect the health of a litter during pregnancy, birth, and the first few weeks of life, which require adequate attention from both the breeder and the veterinarian. Maternal, neonatal, environmental, and birth-related factors can directly influence litter viability [11,12,13]. Furthermore, lack of knowledge, management errors, lack of neonatal care, and neglect also influence neonatal mortality.

For greater prolificity and neonatal survival in kennels and catteries, maternal and neonatal management and care are essential and must be carried out properly by breeders. For this, the veterinarian needs to be updated on the neonatology of small animals to properly instruct breeders and provide neonatal assistance. Neonatal losses are still common but should not be considered normal, and greater knowledge of neonatology will impact neonatal survival in breeding. The main risk factors for neonatal mortality, as well as the care and adjustments that must be carried out for the success of the breeding, are described in detail in this review, which addresses maternal and neonatal care from the veterinarian’s perspective and is intended for veterinarians in the practice of small animals, especially those in contact with dog and cat breeders. The scientific articles used to support this review were selected from the following databases: PubMed/Medline, Scopus, Science Direct, and Google Scholar, based on the search for the keywords: Puppy, kitten, neonatal health, management, breeding, reproduction, mortality, bitch, queen, neonatal care, maternal care.

## 2. Knowledge of Neonatology by the Veterinarian

For many years, in most faculties of veterinary medicine existing in many countries, neonatology was not part of the teaching curriculum. This fact makes it less likely that veterinarians have basic knowledge of neonatal care and particularities. This is probably due to castration campaigns and efforts to control the overpopulation of dogs and cats, resulting in less routine contact between veterinarians and newborns, since most of their patients are gonadotectomized [14]. However, in recent years, there have been changes in relation to pet breeding, such as the growing commercial breeding in kennels and catteries, culminating in a greater number of newborn patients requiring assistance in the clinical routine, and consequently, veterinarians having contact with more newborns throughout their careers. In this way, the teaching of topics on neonatology in the veterinary medical curriculum is essential and has evolved over time [14,15].

The lack of knowledge in neonatology by the veterinarian directly affects the dissemination of correct management information to breeders and the lack of adequate assistance to the sick neonate. In addition, risk factors often go unnoticed or are overlooked. There is a frequent failure in the diagnosis of neonatal affections, leading to a lack of treatment and reduction in prevention, which contributes to a high mortality rate.

Clinical examination, neonatal care, and most affections are markedly different from that of adults due to physiological and hemodynamic differences. In this way, veterinarians are often afraid to evaluate and treat newborn patients due to the small size and presumptive limitations of diagnostic and therapeutic interventions. However, we have the ability to treat newborn animals effectively [6,16]. This fear can lead to the absence of an early diagnosis and adequate treatment that could prevent patient mortality. Therefore, veterinarians must be aware of the unique distinctions between neonate and adult animals regarding normal physiological variables that affect clinical examination and diagnostic test results, as well as treatment [16].

Knowledge about the neonatal period of dogs and cats is therefore essential. The update in the study of neonatology contributes to a better assistance to breeding and medical management. This is an area of veterinary medicine in which having a strong knowledge base of physiology and common clinical presentations for newborns (hypothermia, hypoglycemia and dehydration), in addition to the main affections, can often improve outcomes [14]. The benefit will be evident, and better assistance, diagnoses, treatments, and implementation of preventive measures will reduce mortality rates.

## 3. Knowledge of Neonatal Care by Breeders

The management carried out by breeders and their employees is always an important stage of neonatal care and the survival of patients, since the management of the litter demands attention, patience, and meticulous care for several hours of the day. The breeder must rely on the assistance of a veterinarian in its breeding, and annual training in basic neonatology for employees will lead to proper handling and can maximize the survival of the litters.

For assistance in breeding, some questions should be asked to the breeder, such as the experience he has in relation to the breeding of dogs and cats, with the specific breed, and if he has practice in assisting the birth and general management of litters [15]. In addition, the general management, monitoring, maternal and neonatal care, mortality rates in the breeding, and whether the kennel or cattery facilities are adequate should be evaluated.

Education on the correct management of the breeder and employees must be carried out, emphasizing the particularities and the risk of mortality of these patients. They should be instructed in antenatal care, delivery assistance, and later neonatal care, encouraging them to monitor for early warning signs of newborns that are failing to thrive, such as failure to gain weight [14,17], so that early assistance can be instituted. The correct management of nutrition (feeding, hydration, passive immunity), body and environmental temperature, and health by breeders will influence neonatal health and well-being.

## 4. Pregnancy and Prenatal Care

The health of the fetus and neonate is closely linked to maternal health; in this way, care for neonates begins before birth [18]. The course of a pregnancy fundamentally determines the viability of a newborn during its first weeks of life. The number of factors that can affect the mother’s condition during this period is countless [12]. Age, genetics, nutrition, vaccination status, and the presence of infectious diseases, among other maternal conditions, can directly interfere with litter survival [13,19,20]. A study has shown that these maternal factors can affect more than 30% of kennels [5] and can lead to a 9% mortality rate in newborn dogs [2].

### 4.1. Genetics and Inbreeding

The ideal management for the birth of healthy puppies and kittens starts with choosing the most suitable parents for mating, and genetics is a determining factor for successful reproduction [19]. Rigid selection must be carried out, and lines of bitches and queens with obstetric problems, defects, or congenital malformations, poor maternal ability, low prolificacy, hereditary diseases, and other genetic factors must be avoided, as they will increase the risks of complications and mortality in the litter [19,21,22,23].

Inbreeding is an important factor in losses in kennels and catteries. The main genetic effect of the reproduction of related animals is the loss of genetic variability and the increase in homozygosity due to the high number of genes per descent, causing the manifestation of deleterious recessive alleles. Thus, inbreeding can have a significant impact on the survival and quality of offspring, as well as increasing the predisposition to hereditary diseases. At the same time, a decrease in genetic diversity can be harmful to animals at the individual and species levels [24,25,26,27]. A study has shown that inbreeding can lead to malformations and consequently high mortality in dogs [28]. Furthermore, a high inbreeding rate can result in impaired fertility and therefore a reduction in the number of pups that are born in the litter [29]. It is the role of the veterinarian to make breeders aware of the consequences of performing inbreeding and that this should be avoided.

### 4.2. Vaccination Status and Deworming

Vaccination of females is an important factor to consider before breeding. Unvaccinated females are at increased risk of miscarriage, preterm birth, stillbirth, and neonatal death from infectious diseases. In addition, the deficient vaccination status will influence the quality of colostrum, which will impact the immunity of the puppy and kitten, leaving newborns vulnerable to infections during the neonatal period [20,21]. The veterinarian must instruct the breeder on vaccination protocols for bitches and queens. The female must be referred to the reproductive process, having her vaccination boosted at least one month before reproduction, so that there is minimum time for the effective production of antibodies.

Maternal deworming before and during pregnancy is also an important factor for the health of the litter, given that there may be parasitic transmission via placental, lactogenic, and environmental contamination. It is recommended to deworm pregnant bitches and queens, and from other animals in the same environment, as well as their neonates, to prevent environmental contamination and infection of puppies and kittens [18,19]. In many cases, females do not show clinical signs of parasitic infection, such as diarrhea, but shed eggs in the environment, which will serve as a source of infection for the litter. As newborns are more susceptible, they can develop serious infections, resulting in death. As with vaccine boosters, the female must be properly dewormed prior to mating. Deworming should be performed again at 45 days of gestation and on the day of delivery, suggesting this protocol in pregnancies with fenbendazole [19].

### 4.3. Maternal Age

Maternal age has an influence on reproductive performance; above six years of age, bitch fertility begins to decrease, with a reduction in prolificacy. At five years of age, failure to conceive can be more than 50%. However, the age of reproductive senescence depends on the size of the bitch [20,22,30]. In addition, older bitches and queens may show changes in the estrous cycle, greater muscle flaccidity, and are more predisposed to dystocia and litter losses, among other factors [31].

A significantly higher neonatal mortality rate is observed in neonates of mothers older than six years of age [32]. Older bitches can give birth to weak and debilitated puppies, with a high mortality rate between birth and weaning [22]. Seven-year-old bitches can have a litter mortality of approximately 38.9%, while in 3.5-year-old bitches, the mortality is lower–approximately 15%. In two-year-old bitches, mortality was approximately 18.5% [30]. Another study showed that females younger than two years and older than eight years are more likely to give birth to low-birth-weight pups [17]. These neonates are more immature and predisposed to early mortality. Therefore, very young or old bitches and queens should be avoided in breeding–a fact that must be addressed to breeders.

Maternal age also has a significant influence on neonatal blood glucose. The average glucose concentration of newborns can vary in the first eight hours after birth, according to the age of the mother. Neonate dogs born to bitches under four years of age have a mean glucose concentration of 125 mg/dL. In newborns of bitches between four and six years of age, neonatal blood glucose is on average 97 mg/dL, whereas in bitches over six years of age, the average glucose concentration of puppies is 82 mg/dL [33]. Maternal age is considered a risk factor for lower neonatal survival, as glucose concentrations below 92 mg/dL in newborns are correlated with high mortality during the neonatal period [33].

### 4.4. Nutrition

The nutritional status of the pregnant female can interfere with the health of puppies and kittens. The newborn depends almost exclusively on liver glycogen for energy during the first 24 h. Liver glycogen stores may be reduced at birth due to maternal malnutrition [19,21], resulting in a greater predisposition to neonatal hypoglycemia. Females with poor nutritional status will give birth to weak neonates and produce poor-quality colostrum, resulting in higher mortality rates. On the other hand, maternal obesity reduces prolificacy, and obese bitches and queens have a higher risk of dystocia and higher rates of perinatal mortality [19,31]. Therefore, the body score should always be evaluated prior to referral to the reproductive process, avoiding malnourished, thin, or overweight females.

In addition, errors in the nutritional management of pregnant and lactating can trigger diseases in puppies and kittens. Excess vitamin A is related to fetal mummification, birth of weak neonates, and malformations such as cleft palate (palatoschisis), twisted tail, ear deformities, and nervous system malformations. Vitamin D excess has been associated with early fontanelle closure, tissue calcinosis, enamel hypoplasia, and supravalvular stenosis [19,31].

Feeding with excess calcium or protein in pregnant and lactating females may be associated with puerperal hypocalcemia (due to reduced parathyroid hormone stimulation) and swimmer puppy syndrome (myofibrillar hypoplasia), respectively. Diets with a nutritional deficiency of vitamin K are related to hemorrhagic syndrome in neonates [19,34]. In queens, nutritional deficiency of taurine can result in embryo resorption, miscarriage, and stillbirth [19]. Excessive maternal supplements should be avoided. A quality diet suitable for pregnancy and lactation will be sufficient for adequate maintenance of pregnancy and the health of the female.

Metabolic disorders such as diabetes mellitus in the bitch have been associated with fetal loss due to persistent hyperglycemia and the development of large puppies with a higher risk of fetal dystocia. Other metabolic abnormalities, such as hypothyroidism, puerperal hypocalcemia, and pregnancy toxemia, can result in fetal or neonatal loss [13,35]. In addition, prenatal and postnatal nutrition contribute to the metabolic programming of the puppies and kittens. Maternal nutrients can influence fetal gene expression, interfering with the animal’s metabolic state for life [19].

### 4.5. Behavior and Maternal Instinct

Maternal behavioral factors after parturition or during lactation also influence litter health. The failure of maternal instinct is a risk factor for mortality in neonates. Primiparous or stressed females are more predisposed to develop this behavior of rejection or aggression toward neonates [13,36,37,38], as well as females submitted to cesarean section without triggering signs of labor, in recovery from anesthesia or sickness [19,39].

Changes in maternal behavior can lead to inadequate breastfeeding in approximately 25% of kennels [5]. Insufficient colostrum intake in the first 12 h of life results in failure to transfer passive immunity, making newborns vulnerable to various infectious diseases [36,39]. In addition, neonates who are not adequately breastfed will develop failure to gain weight, the neonatal triad (hypothermia, hypoglycemia, and dehydration) and fading, progressing to death [11,13,36,37]. On the other hand, females with deficient and aggressive maternal instincts can cause trauma and lacerations in newborns.

Failure in maternal instinct can occur in more than 50% of kennels [5], temporarily or persistently. Therefore, breeders must be aware of maternal behavior to assist newborns when necessary. As a preventive measure, females with a history of inappropriate behavior or poor maternal ability should not be selected for breeding.

### 4.6. Maternal Agalactia or Hypogalactia

Maternal agalactia or hypogalactia are associated with several factors and may occur temporarily in cases of bitches and queens undergoing a cesarean section, primiparous, or under stressful conditions [19]. However, even if it occurs temporarily, it can lead to the manifestation of the neonatal triad, weight loss, fading, and death in the litter. In addition, it can interfere with the absorption of immunoglobulins from colostrum, as the highest rate of absorption by the newborn occurs in the first 8–12 h of birth [40]. On the other hand, some females can present permanent agalactia, and this situation is associated with disorders in the mammary glands, unresponsiveness to physiological stimuli, concomitant illness, hypocalcemia, preterm birth, and malnutrition [15,37].

It is important to instruct breeders to assess females at whelping for milk production. In cases of agalactia or hypogalactia, maternal care with lactogenesis-stimulating drugs should be started immediately, and the institution of nutritional management of neonates must be carried out and maintained until the mother produces milk. A source of passive immunity and breast milk substitute must be provided, ensuring the survival of the litter.

### 4.7. The Importance of Clinical Evaluation and Prenatal Examinations

Prenatal care is the monitoring and essential care of the pregnant female by the veterinarian, aiming to ensure maternal and fetal health and to diagnose and treat possible complications. It consists of performing clinical evaluations and periodic examinations, which are essential before the reproductive process and during pregnancy. In addition, the breeder must be instructed on proper handling during this period.

Regular examinations and monitoring of pregnancy are key tools for the early detection of signs that may indicate disorders even before clinical signs occur. Early detection significantly increases the chances of adequate development and survival of neonates [12].

Bitches and queens that do not undergo a complete clinical examination and complementary exams before and during pregnancy have greater losses in litters [2,19]. The history, thorough physical examination, blood counts, biochemicals, specific tests for infectious diseases (such as herpesvirus, brucellosis, feline viral leukemia virus, feline viral immunodeficiency, among others), ultrasound, radiography, and other complementary exams can determine maternal health, the female’s possibility of entering the reproductive process, and alterations and affections during pregnancy and parturition. The veterinarian needs to be aware of the physiological distinctions of the pregnant female, and the interpretation of complementary exams must be carried out according to the reference parameters of the gestational period. It is essential to emphasize to breeders the importance of prenatal care for the success of breeding.

## 5. Fetal-Neonatal Transition

Birth is a critical period of adaptation to extrauterine life and a major challenge for neonatal survival [41,42]. In the fetal–neonatal transition, the newborn undergoes several physiological changes. Among them, gas exchange is no longer carried out by the placenta and is carried out by pulmonary respiration. The efficiency of gas exchange will depend on adequate respiratory adaptation at birth [33,41,43].

However, prolonged and dystocic deliveries can lead to pronounced asphyxia and severe hypoxia in newborns, leading to failure of respiratory adaptation and higher mortality rates in the transition period [3,11,44]. This condition is considered the main cause of more than 60% of losses of canine and feline neonates during the first two days of life (early mortality) [10,20,45].

Although puppies and kittens can present physiological hypoxia, demonstrating hypercapnia and mixed acidosis during parturition and immediately after birth, this is transient [46,47,48,49,50]. This occurs because the fetus undergoes a short period of asphyxia simultaneous with uterine contractions, which culminates in a decrease in uterine blood flow, placental perfusion, and gas exchange [20,48]. Adaptation to pulmonary breathing after birth is essential for CO_2_ elimination and acid–base balance recovery [46,47,48,49,50]. However, any form of dystocia can worsen asphyxia, resulting in excessive hypoxia and leading to high mortality rates in the first few days after birth [10,20].

Depending on the obstetrical condition or type of delivery, there will be consequences for the neonatal viability and subsequent health of the puppies and kittens. The mortality of canine and feline neonates after severe hypoxia is often influenced by maternal and fetal dystocia [3,11,20,23,44]. Deaths from dystocic births can affect approximately 42% of kennels [5]. In addition, premature, low-birth-weight, and malformed puppies and kittens may not be able to adapt to breathing, requiring immediate assistance.

Neonates who experience prolonged asphyxia and consequent excessive hypoxia may be born in a state of respiratory distress, with bradycardia, cyanosis, dyspnea, bradypnea [7,10,20], pronounced acidosis, and damage to tissues with high oxygen requirements, such as the heart [7,20], as a result of failure in tissue oxygen delivery. Approximately 80% of newborns of dogs and cats with this condition may die during delivery or within the first 24 h after birth [2,10], especially when adequate care for these patients is not provided.

It is essential that deliveries in kennels and catteries are assisted by a veterinarian. Maternal and neonatal clinical evaluation, complementary exams (hormonal, blood glucose, ultrasound, radiography, among others), and the assessment of viability and vitality scores at birth, such as the Apgar score, can diagnose patients at risk and determine the need for immediate intervention, increasing the chance of litter survival [4,10,13,41,42,49,50,51,52,53,54]. The veterinarian must be prepared to intervene quickly in cases of asphyxiated puppies and kittens, knowing and correctly performing the procedures for resuscitating dogs and cats at birth. Immediate care focuses on providing heat to the newborn and on ventilatory and circulatory support, maintaining patent airways and adequate tissue perfusion [11,13,14,41,55,56].

## 6. The Neonate

### 6.1. Adaptation to Extrauterine Life

During the fetal–neonatal transition, the newborn will have to adapt to the various physiological changes that occur to survive to extrauterine life [18,41]. The newborn must not only initiate and maintain adequate lung breathing but also maintain stable blood glucose and body temperature, as well as adapt to the immaturity of the various organic systems, which makes it extremely vulnerable to various changes and effects during its development [39].

Respiratory, cardiovascular, and metabolic physiological changes define the fetal–neonatal transition. This is a critical and high-risk period for mortality [18,48], failure to adapt to extrauterine life is responsible for pup mortality in approximately 34.3% of kennels [5]. Pulmonary changes, such as failure to absorb and replace alveolar fluid with air, surfactant deficiency, pulmonary atelectasis, lack of consistent breathing, and inadequate changes in blood flow, temperature, and energy metabolism, are some adaptive failures that can lead to immediate postpartum mortality [33,43].

It is essential that the veterinarian understands the physiological particularities of the transition period to identify early changes that can lead to failure to adapt to extrauterine life. Vital parameters such as heart and respiratory rate, beyond body temperature, reflexes, tone, breastfeeding, blood glucose, and weight gain of puppies and kittens should be monitored. The constant monitoring of the litter by the veterinarian and the breeder allows an immediate intervention in intercurrences, which ensures survival.

### 6.2. Physiological Immaturity

Canine and feline neonates are born immature in several organ systems, having unique characteristics that completely differ from an adult animal. The physiological particularities of the newborn make it extremely vulnerable to various pathological disorders during its development. The neonatal mortality rate due to changes associated with physiological immaturity can reach approximately 30% of puppies and kittens [21]. Some of the main consequences of physiological immaturity correspond to the neonatal triad (hypothermia, hypoglycemia, and dehydration).

Several physiological aspects are related to the predisposition of the neonate to develop hypothermia, such as immaturity of the hypothalamus, reduced adipose tissue, undeveloped vasoconstriction mechanisms, complete inability of the tremor reflex, inability to pant, and large surface area in relation to body mass [11,17,18,43]. Moderate hypothermia can lead to lethargy, reduced metabolism, inappetence, and decreased neonatal reflexes. However, the newborn still tries to breastfeed, but the milk may not be digested due to reduced motility and consequent intestinal paralysis, which can cause regurgitation, aspiration pneumonia, gas production, and gastrointestinal dilation [11,16,17]. In addition, the ability of lymphocytes to transform and fight infection is significantly reduced when neonates are hypothermic [15]. In severe hypothermia, neonatal clinical depression occurs. The newborns become extremely lethargic, interrupting their attempt to breastfeed, and there is a decrease in cardiorespiratory function, resulting in tissue hypoxia, acidosis, and death [11,17].

Some features make neonates particularly susceptible to hypoglycemia. Due to liver immaturity, neonates are born with limited glycogen stores and minimal capacity for gluconeogenesis [11,16]. In puppies and kittens that are not suckling, blood glucose can drop rapidly, as the ability to maintain normoglycemia in cases of fasting is reduced. Liver reserves will be completely depleted within 24 h [16,20]; however, a blood glucose decline may occur before this time in frail, sick, premature, or low-birth-weight neonates. Hypoglycemic neonates may manifest crying, weakness, clinical depression, reduction or absence of the sucking reflex, and interruption of breastfeeding, aggravating the condition. Severe hypoglycemia can lead to bradycardia, seizures, coma, and death [15,16,17]. Blood glucose is a predictor of mortality in neonates, and glucose concentrations < 92 mg/dL in the first 24 h of life increase the risk of death during the neonatal period [33].

Newborn puppies and kittens are still essentially more susceptible to dehydration than adult animals, mainly due to renal immaturity, presenting a lower capacity to conserve water. In addition, other physiological particularities, such as a higher concentration of body water, a large proportion of surface in relation to body mass, and greater fluid loss through immature skin, increase the predisposition of neonates to dehydration [11,16,20]. Dehydrated neonates can progress to hypovolemia, hypotension, shock, and death [11,20].

It is important to point out that any neonatal alteration or disease can lead to clinical depression, apathy, reduction in the sucking reflex, and consequent reduction in milk intake, which can lead to the neonatal triad [7]. In addition, maternal alterations, such as agalactia, hypogalactia, failure of the maternal instinct, and errors in management, can cause the newborn to manifest hypoglycemia, hypothermia, and dehydration.

Although physiological immaturity represents a challenge for neonatal survival, it is important to emphasize that newborn patients are able to grow up healthy under adequate management and health conditions [16]; for example, the correct management of nutrition (adequate breastfeeding, hydration, source of passive immunity) and the environment (adequate temperature, humidity and hygiene) can prevent the manifestation of the triad and guarantee the adequate development of the litter.

### 6.3. Passive Immunity Transfer Failure

The immune system of newborn dogs and cats is relatively immature and slowly reactive; thus, newborns’ immune response to various pathogens is lower than that of adult animals, making them susceptible to infections [57,58,59]. In addition, minute amounts of immunoglobulins can be transferred transplacentally to developing fetuses, approximately 5–10% in dogs and up to 25% in cats, compared to circulating titers in adult animals [15]. Thus, to maintain adequate immunity during the first weeks of life, neonates are completely dependent on the passive transfer of antibodies through colostrum [36,39,58,59].

Inadequate colostrum intake results in failure to transfer passive immunity, which is associated with high mortality rates during the neonatal period [36,60]. The prevalence of failure to transfer passive immunity in dogs is high, approximately 17.4% [36]. The risk of death from birth to 21 days of age is higher if the serum IgG concentration is below 2.3 g/L compared to puppies with higher concentrations. Approximately 40% of puppies with IgG concentrations below the threshold ≤ 2.3 g/L will die during the neonatal period [36].

The achievement of passive immunity by the neonate will depend on three main factors: the amount of colostrum ingested (which will depend on the milk production by the female, on the maternal behavior toward breastfeeding, and on the presence of the neonatal sucking reflex), the immunological quality of colostrum (i.e., its immunoglobulin concentration), and the intestinal barrier closure (ability of the neonatal digestive mucosa to absorb ingested antibodies) [39]. Closure of the intestinal barrier in the canine species begins 4–8 h after birth and is completed within 16–24 h [40]. However, after the first 12–16 h of life, the rate of IgG absorption is practically absent [39,40]. Thus, it is essential to ensure that the newborn has colostrum intake in the first 12 h of life, and when there is a deficit in breastfeeding and colostrum intake is not possible, a colostrum substitute should be of/fered (natural or artificial hyperimmune solutions), such as colostrum bank, commercially available colostrum substitutes, supplements based on hyperimmunized powdered egg and serum, or blood plasma from a healthy and vaccinated animal of the same species [33].

It is important that the female is evaluated for colostrum production and her behavior to breastfeed, and that neonates are evaluated for sucking force for suckling and weight gain. The assessment of weight gain, from birth to two days of age, is highly correlated with the concentration of serum IgG in the neonate, as colostrum, in addition to immunoglobulins, provides energy for the growth of the newborn [36,61]. Newborns should gain at least 5–10% of their body weight per day [15]. In a study by Mila et al. [62], the growth rate in the first two days of life below 2.7% allowed the identification of the transfer deficit of passive immunity in 87–96% of cases.

### 6.4. Low Birth Weight

Neonatal health is affected not only by factors after birth but also by fetal life during pregnancy. Adequate growth as a fetus guarantees the neonate the maturity necessary for birth and its ability to survive extrauterine life [39].

Low birth weight neonates are considered a risk group for mortality [39,61,62]. These newborns suffered intrauterine growth restriction due to the deprivation of adequate oxygenation and nutrition [61]. This condition can occur due to maternal and fetal factors and placental changes, affecting the supply to the fetus [17,32,38,63], as well as competition for uterine blood support in large litters [17,32,38]. In addition, female neonates were also associated with a higher risk of low birth weight [32]. The consequences of this will be neonates who manifest clinically low birth weight and who are physiologically more immature than littermates of average breed weight at birth [11,32,64].

Due to immaturity, low-birth-weight neonates are susceptible to several risk factors for mortality, demonstrate lower viability at birth, and may be born weakly and debilitated, with lower vitality scores, immunity deficits, lower liver glycogen stores, and greater deficiency of the thermoregulatory system [11,17,32,33]. These newborns may also have a weak or absent sucking reflex or may not be able to compete for breastfeeding with larger and more robust newborns in the litter [11,64]. In view of this, these newborns are even more predisposed to possible complications of neonatal vulnerabilities, such as the neonatal triad, fading syndrome, and infections [11,17,32].

Weight is of high relevance in predicting neonatal mortality; more than 80% of puppies that die within the first 48 h of life (early mortality) are low-birth-weight neonates [61], and approximately 60% of low-birth-weight kittens do not survive to weaning [15]. A study in dogs showed that mortality rates were 4.2, 8.8, and 55.3% in puppies born normal, low birth weight, and very low birth weight, respectively [32]. Birth weight must be analyzed according to each breed due to the large variation in body weight between them [62]. Thus, neonates born 25% lighter than the average weight of their littermates, or their breed, have a higher risk of death [39]. Table 1 shows the average adequate weight of dogs and cats at birth according to breed size.

Surprisingly, a study has shown a future consequence of this condition: dogs with low birth weight are more likely to become obese in adulthood, similar to humans. This may be due to the restricted energy supply, and the offspring may develop metabolic adaptations early in life to promote survival [65].

Knowing that adequate weight is a reflection of the relative maturity of the organism and health in neonates [32,64] and that low birth weight is an important risk factor in neonatal mortality, it is imperative that smaller newborns have special care [14,66], including assisted breastfeeding or supplementation, and accurate recording of weight gain. Colostrum intake should be ensured, breastfeeding monitored, milk supplementation instituted in puppies and kittens with failure to gain weight, orogastric tube feeding implemented for neonates with inadequate suction, and maintenance of an adequate body and environmental temperature undertaken. Assistance and monitoring by breeders are essential to prevent the fading and mortality of newborns.

A study on low-birth-weight neonates demonstrated that management and care practices by dog and cat breeders, such as weighing newborns, monitoring breastfeeding or artificial feeding, can have a beneficial impact on the survival of these high-risk neonates [67].

### 6.5. Congenital Malformations

Congenital malformations are functional or structural anomalies in organs or systems during fetal development that can interfere with the viability and health of newborns [15].

These birth defects have high mortality rates during the neonatal period [28]. The incidence of congenital malformations in dogs is 6.7%, and approximately 68% of these newborns can die [28]. In cats, birth defects occur in 5% of newborns [8].

The malformations may be present in more than one animal in the litter and may result in fetal and neonatal death or even in euthanasia due to defects incompatible with life. The overall prevalence of litters containing one or more neonates with birth defects is relatively high, 24.7% in dogs [28] and 14.3% in cats [67].

The manifestation of malformations in puppies and kittens is related to genetic factors or maternal exposure to teratogenic agents during pregnancy. Genetic defects can be hereditary, being more common in purebred dogs and cats, or even manifest due to the consequences of inbreeding. Some dog breeds manifest malformations relatively frequently, such as Pug, French Bulldog, English Bulldog, American Bully, German Spitz, Miniature Pinscher, Pitbull, Shih-tzu, and Yorkshire [15,28]. Teratogenic agents, such as nutritional imbalance (excess of vitamin A, D, and proteins), drugs (tetracyclines, fluoroquinolones, steroids, among others), toxins, infectious diseases, mechanical influences, and irradiation, can affect litter during gestational development [19,21,68].

The most frequently observed malformations in dogs are cleft palate (2.8% incidence) and hydrocephalus (1.5% incidence), which can generate mortality in approximately 90% and 40% of those affected, respectively [28]. Other diverse malformations are described in dogs and cats, such as anasarca, cleft lip, pectus excavantum, gastroschisis, omphalocele, exencephaly, cardiac vascular anomalies, anal atresial, swimming puppy syndrome (myofibrillar hypoplasia), and rectovaginal fistula, among others [11,15,19,21,28,34].

At birth, all neonates in the litter must be carefully examined for the presence of malformations, and the breeder must also be oriented and taught how to assess the presence of defects in the neonate. Although many malformations are identifiable by physical examination, many go unnoticed due to failure in neonatal assessment, which contributes to a high mortality rate. Other defects will only be diagnosed after complementary exams or postmortem examination. Thus, in any mortality without apparent cause in the litter, a necropsy should be performed [21,28,69]. It is important to note that many malformations occur in an associated way. The newborn may have more than one congenital defect, external or internal; in view of this, even after the diagnosis of a defect, the possible presence of more malformations should not be ruled out.

Some internal malformations may not yet trigger the manifestation of clinical signs during the neonatal period and may be more commonly diagnosed only in the juvenile phase or in adult life, such as congenital heart disease and congenital metabolic disorders. Many will have medical or surgical management and treatment [18,28,34,69], while others are incompatible with the life of the newborn. However, birth defects should not be neglected, as not all will be cases of euthanasia. It is important for the veterinarian to be aware of the clinical approach to malformations to carry out the procedure properly, as well as to explain to the breeder the possibility of handling/treating each defect. Each case must be evaluated individually.

Prevention will always be the best option. Care must be taken when choosing parents for breeding, avoiding parents with a history of genetic problems or defects, as most malformations in dogs can be related to genetic factors–the highest incidence (84.4%) is observed in purebred dogs [28]. In addition, maternal exposure to teratogenic factors during pregnancy should be avoided. Prenatal care is essential to prevent malformations and reduce mortality rates in litters of dogs and cats.

### 6.6. Neonatal Infections

Infection in kennels and catteries is highly relevant to perinatal mortality, with the mother and the environment being the most common sources of infection in the litter. Many infectious agents are transmitted in the uterus, through milk and other maternal secretions, through contact with other animals, and through the contaminated environment [7,34,70]. Perinatal infections by bacterial, viral, and parasitic agents can cause embryonic or fetal death, miscarriage, stillbirth, neonatal mortality, and the birth of weak, debilitated, and septic neonates [7,15,34,71].

During the perinatal period, puppies and kittens are commonly at increased risk of contracting various infectious diseases, as the immune system is not fully developed. Morbidity and mortality rates from bacterial, viral, and parasitic infections are essentially higher than in adults [15,21].

Among these, bacterial infections are the most commonly observed in the neonatal period [11], affecting 34% of dogs during the first four weeks of life [7]. In approximately 65% of newborn pups, mortality can be attributed to a bacterial infection [70]. Sepsis is the leading cause of mortality in dogs during the first three weeks after birth [13,70,72].

The incidence of neonatal sepsis in dogs is high, affecting approximately 14.8% of newborns, and the mortality rate is approximately 25.6%, which is highest during the first days of life. Early mortality (0 to 2 days of age) can reach 69% of affected newborns [7].

Neonates will be more predisposed to infections if colostrum is not ingested soon after birth, due to the failure to transfer passive immunity [11]. In addition, poor environmental hygiene predisposes to the survival of several pathogens that can threaten the lives of puppies and kittens [15].

The main bacterial agents involved in neonatal infections are *Escherichia coli*, *Staphylococcus* spp., *Streptococcus* spp., *Klebsiella* spp., *Enterococcus* spp., *Pseudomonas aeruginosa*, *Chlamydia psittaci*, *Ureaplasma* spp., *Proteus* spp., *Salmonella* spp., *Klebsiella* spp., *Mannheimia hemolytica*, *Pasteurella multocida*, *Enterobacter* spp., and *Brucella* spp. [7,20,34,70].

Bitches and queens may not show clinical signs; often, the infection is restricted to the uterus, with no systemic changes. However, infectious agents can have fatal effects on fetuses or newborns [7,34,64,72]. In many cases, neonatal infection can go unnoticed if a thorough clinical evaluation of the newborn is not performed and, as a result, litter mortality can occur.

The clinical manifestations of sepsis in newborn dogs vary considerably, and many can be nonspecific, such as diarrhea, weight loss and the neonatal triad, which are common to several changes or diseases in the newborn. However, neonates may manifest more specific clinical signs of infection/sepsis, such as body hyperemia, omphalitis, abdominal bruising, and cyanosis or necrosis of the extremities of limbs, tail, or ear [7]. It is important that the veterinarian instructs the breeders to monitor the litters in search of these clinical signs, and if any of them is noticed, quickly forward them to specialized care.

Due to immunological and other organ system immaturity, neonatal sepsis can have a hyperacute course and high mortality in litter [20,72]. However, it is possible to identify clinical signs of neonatal infections early. The newborns’ chances of survival will be greater if sepsis is diagnosed quickly and treatment is instituted as soon as possible. Therefore, constant monitoring of the litter is essential in breeding.

The accomplishment of the leukogram followed by the bacterial culture are important complementary exams in the diagnosis of neonatal sepsis. Therapy for affected neonates involves the selection of antibiotics that are broad-spectrum and safe for neonates. Antimicrobials of the cephalosporin and penicillin classes are recommended in neonatal therapy, as they can be used safely in these patients [7,18,72,73]

Regarding parasitic and viral infections, the incidence is also high; approximately 77% of kennel puppies are infected by at least one enteropathogen, with 29.3% of them being able to excrete three or more pathogens [74].

Parasitic infections in neonates can occur in up to 60% of kennels and catteries [21]. Several pathogens are involved in internal parasitic infections of newborn dogs and cats. The nematodes *Toxocara* spp., *Ancylostoma* spp., *Strongyloides* spp., *Trichuris vulpis*, the cestode *Dipylidium caninum,* and the protozoa *Giardia* spp., *e Cystoisospora* spp. have high rates of infection and can cause high mortality in litters [11,13,21,75]. They can lead to clinical depression, lethargy, reduced or absent reflexes, neonatal triad, diarrhea, constipation, dehydration, hypoproteinemia, vomiting, anemia, hematochezia, and in cases of massive nematode infection, intestinal obstruction, intussusception, intestinal perforation, and peritonitis [11,15,21,74,75].

Furthermore, ectoparasites present on the mother, such as fleas and ticks, can quickly migrate to newborns through maternal contact and the environment. As they are hematophagous, neonatal infection can result in anemia [13,76,77] in addition to the risk of infection by other pathogens to which ticks and fleas are vectors, such as *Babesia* spp., *Ehrlichia* spp., and *Dipylidium caninum* [15,75].

The effectiveness of management strategies to prevent parasitic infections in kennels and catteries will depend on knowledge about the characteristics of the parasites and their life cycle [75]. Environmental cleaning and disinfection with suitable products are essential. Prenatal care, such as performing coproparasitological exams before reproduction, deworming the mother and other animals in the environment, and preventing ectoparasites in pregnancy, reduces the possibility of parasitic infections in the litter. Newborns should also be dewormed and constantly monitored, as the evolution of the disease can occur quickly in parasitized neonates, and early diagnosis and treatment will be essential in these patients.

Viral infections are usually more common after weaning due to decreasing protection by colostrum antibodies over time. However, they can occur in the perinatal period, and maternal infections and neonates with colostrum intake deficiency and low passive immunity can develop viral infections early during the first weeks of life, which can lead to high mortality rates [11]. The main agents of viral infections in dogs that affect puppies include canine herpesvirus type 1, canine parvovirus types 1 and 2, canine distemper, canine adenovirus types 1 and 2, and parainfluenza virus [15,74,77]. In kittens, feline herpesvirus type 1, feline calicivirus, feline parvovirus, feline viral leukemia virus, and feline immunodeficiency virus are often associated with litter losses [11,13,15,74,77].

In general, these viruses can induce embryo resorption, abortion, preterm birth, and stillbirth. In addition, the birth of debilitated neonates, which may manifest clinical depression, inappetence, neonatal triad, and gastrointestinal, respiratory, or neurological disorders, can result in early mortality in the litter [15,21,77].

The prenatal evaluation and the performance of specific tests for viral infections in bitches and queens before reproduction will prevent the mating of sick females or carriers of these infectious agents, reducing the risk of perinatal mortality. Vaccination before reproduction is an essential measure to ensure maternal immunity against various infectious agents and the health of the litter. In addition, vaccination ensures adequate levels of immunoglobulins in colostrum, which will be vital in protecting newborns.

Neonatal mortality in puppies and kittens is a problem frequently encountered by dog and cat breeders. Often, postmortem examination allows determination of the cause of miscarriage, stillbirth, or neonatal death, which is essential for the implementation of measures to save the rest of the litter and for the prevention of risk factors in future breeding litters [78].

## 7. The Environment

Environmental management is an important factor in maintaining maternal and neonatal health and well-being. Mothers and newborns need a comfortable, dry environment, with adequate bedding and controlled temperature and humidity, that is constantly sanitized and free from drafts and stress factors. Errors in environmental management may be responsible for high mortality rates during the neonatal period [5,11].

### 7.1. Maternity Location

The female should be introduced to the maternity place approximately two weeks before parturition so that she gradually adapts, feels comfortable, and starts building her nest [79]. The place should be a quiet environment, free of sounds, and without the constant traffic of people and animals [15].

Stress related to inadequate location can be responsible for considerable neonatal mortality rates [13,15]. Constant stress can depress the immune system, increasing the risk of infections [15]. Pregnant females that are subjected to environmental stress may experience fetal loss [15,79], while stressed lactating females may develop reduced milk production (hypogalactia or agalactia) and behavioral disorders such as cannibalism and failure of the maternal instinct, leading to anxiety and abandonment of newborns [15,21]. Maternal stress is a condition that contributes to neonatal mortality in approximately 8.6% of kennels [5].

In addition, the inappropriate location of the maternity unit, such as in places with open doors or windows, can still allow air currents to pass through, which can lead to hypothermia in newborns [15]. The mother may reject or ignore cold neonates, further aggravating the condition [18,21].

### 7.2. Temperature and Humidity

Environmental temperature control is essential for neonatal survival. Neonate dogs are immature in the thermoregulatory system and are not able to maintain an adequate body temperature without maternal heating or an environmental heat source [13,14,18]. In the presence of the mother, the comfortable ambient temperature in the maternity ward for the newborns and for the mother is 20 to 24 °C. In the absence of the mother, the ambient temperature must be corrected, as the neonates will be without the mother to warm them. The ideal ambient temperature will depend on the age of the neonates, and it should remain between 29 and 32 °C in the first week. This temperature should be gradually reduced over the weeks, between 26.7 and 29.4 °C in the second and third weeks and between 21 and 24 °C in the fourth week [14,18,21].

The absence of heating is a risk factor for neonatal mortality, and approximately 28% of kennels lose puppies due to hypothermia [5]. Incorrect ambient temperatures can interfere with neonatal vitality, leading to depression and lethargy. Hypothermia significantly reduces neonatal metabolism, which leads to bradycardia and depresses gastrointestinal and immune function. Sucking capacity and intestinal motility are reduced, which impairs milk intake and digestion, as well as nutrient absorption. In the first hours of birth, hypothermia also impairs the absorption of colostrum immunoglobulins [15,18].

On the other hand, very high temperatures should be avoided. Hyperthermic environments can stress the mother, who may not feel comfortable for lying down and caring for her neonates [15]. High temperatures can cause dehydration and constipation in newborns. In addition, an overheated environment causes respiratory distress, predisposing patients to respiratory failure [20].

It is important for breeders to be careful with sources of ambient heating, especially when using lamps, thermal mattresses, and electric heaters, so as not to overheat or burn newborns [14]. Breeders should be advised that the maternity ward should have a less heated escape area so that the neonates can move around in case of excessive heat. Another important factor is the ambient humidity, which must be maintained between 50 and 60% [18,21]. High humidity accompanied by high temperature increases the chance of bacterial growth, as well as the appearance of serious respiratory conditions. However, very low humidity can cause excessive dryness of the skin and mucous membranes and dehydration [15,21]. The control of ambient temperature and humidity is essential for neonatal health [13] and can be performed with the use of a thermohydrometer in the place where the litter is.

### 7.3. Bed

The place where neonates will stay can be a specific maternity box or an adapted box [79]. A poorly designed maternity crate can lead to litter mortality. The box must not be small, as there is a risk of the mother causing trauma or suffocating the newborns, stepping on them, or lying on them. On the other hand, the box should not be large either, as there is a greater chance that neonates will separate from the mother and become hypothermic [15]. For small and medium-sized bitches and queens, the maternity ward can be a plastic box or a basket, while for large breeds bitches, the ideal is a specific maternity crate, with side bars that prevent the mother from lying on the newborns [80].

The material used in the bedding must be soft, easy to clean and must not retain moisture. The use of inappropriate material can lead the newborn to become tangled or lost, unable to approach the mother to breastfeed or to keep warm. Nonabsorbent materials can cause neonates to be constantly on a wet surface, with a risk of developing hypothermia [15,21]. If newborns are in a bed damp or wet with birth fluids, for example, they quickly lose heat and become hypothermic. Furthermore, if neonates are placed near cold objects, such as cage floors, they will lose considerable amounts of heat [15].

Plastic boxes are great nests, as they are easy to clean and dissipate less heat. Regardless of the type of material used in the bedding, it must be kept clean and changed frequently [15,21].

### 7.4. Environmetal Hygiene

Adequate environmental hygiene is essential, newborns are more vulnerable to infectious agents due to the immaturity of the immune system. Thus, the absence of environmental cleaning and disinfection is associated with high mortality in the neonatal period. Proper cleaning must be carried out with water and neutral soap, and soon after, disinfection must be carried out with efficient and specific disinfectants against various environmental pathogens. It is important to be careful in the selection of disinfectants and in the chosen concentration as they can become toxic to neonates because the newborn’s skin is thinner, and thus absorption is faster than in adults. In addition, several products can irritate the respiratory system of neonates. Caution should be exercised with the use of phenols, sodium hypochlorite, and other disinfectants, as well as the use of high concentrations [15].

## 8. Conclusions

The perinatal period in dogs and cats has high mortality rates, and the causes of death and risk factors are often not investigated. At the same time, the veterinarian’s lack of knowledge about the main triggering causes can result in failure to diagnose and provide adequate care for these patients.

It is essential that the veterinarian understands the main aspects involved in the management of puppies and kittens and that he has basic knowledge about physiology, prenatal and neonatal care, and preventive strategies. The instruction to breeders on the correct handling and the annual training of their employees allows for better conduct, impacting greater neonatal survival and the success of breeding.

## Figures and Tables

**Table 1:** Mean birth weight of dogs and cats [15,21].

Cats	90–120 g
Small breed dogs	100–200 g
Medium breed dogs	250–350 g
Large breed dogs	350–500 g
Giant breed dogs	600–700 g

## Data Availability

Not applicable.
